# Neuroanatomical anomalies associated with rare *AP4E1* mutations in people who stutter

**DOI:** 10.1093/braincomms/fcab266

**Published:** 2021-11-13

**Authors:** Ho Ming Chow, Hua Li, Siyuan Liu, Carlos Frigerio-Domingues, Dennis Drayna

**Affiliations:** 1 Department of Communication Sciences and Disorders, University of Delaware, Newark, DE 19713, USA; 2 Katzin Diagnostic & Research PET/MR Center, Nemours/Alfred duPont Hospital for Children, Wilmington, DE 19803, USA; 3 Department of Psychiatry, University of Michigan, Ann Arbor, MI 48109, USA; 4 Section on Genetics of Communication Disorders, NIDCD/NIH, Bethesda, MD 20892, USA; 5 Section on Developmental Neurogenomics, NIMH/NIH, Bethesda, MD 20892, USA

**Keywords:** voxel-based morphometry, fractional anisotropy, gene expression, corpus callosum, thalamus

## Abstract

Developmental stuttering is a common speech disorder with strong genetic underpinnings. Recently, stuttering has been associated with mutations in genes involved in lysosomal enzyme trafficking. However, how these mutations affect the brains of people who stutter remains largely unknown. In this study, we compared grey matter volume and white matter fractional anisotropy between a unique group of seven subjects who stutter and carry the same rare heterozygous *AP4E1* coding mutations and seven unrelated controls without such variants. The carriers of the *AP4E1* mutations are members of a large Cameroonian family in which the association between *AP4E1* and persistent stuttering was previously identified. Compared to controls, mutation carriers showed reduced grey matter volume in the thalamus, visual areas and the posterior cingulate cortex. Moreover, reduced fractional anisotropy was observed in the corpus callosum, consistent with the results of previous neuroimaging studies of people who stutter with unknown genetic backgrounds. Analysis of gene expression data showed that these structural differences appeared at the locations in which expression of *AP4E1* is relatively high. Moreover, the pattern of grey matter volume differences was significantly associated with *AP4E1* expression across the left supratentorial regions. This spatial congruency further supports the connection between *AP4E1* mutations and the observed structural differences.

## Teaser

While gene mutations associated with stuttering have begun to be identified, their effects on human brains remain largely unknown. Chow et al. report that stuttering mutations in the *AP4E1* gene are associated with structural differences in the corpus callosum and the thalamus, linking genetics and brain anomalies in stuttering.

## Graphical Abstract

**Graphical Abstract:**
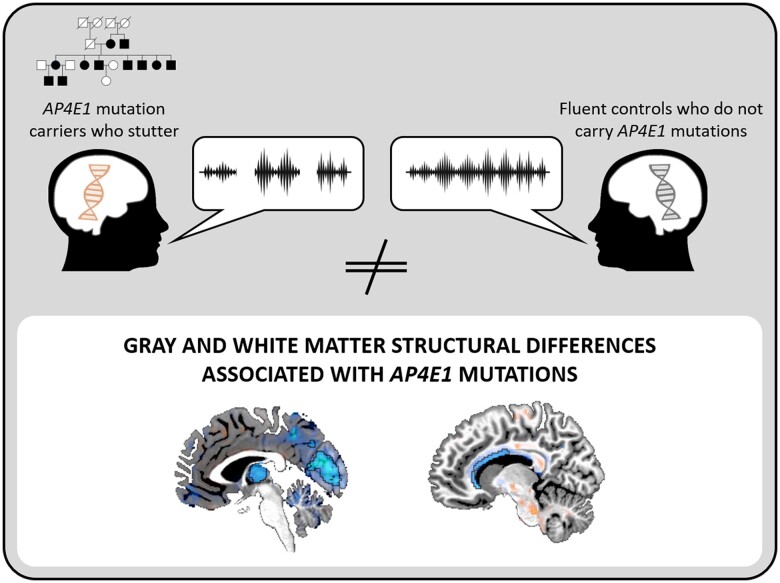

## Introduction

Developmental stuttering is one of the most common speech disorders, affecting about 5% of children and 1% of adults. It is a highly heritable disorder and likely to be a complex polygenic trait.^1^ Recently, persistent stuttering has been associated with mutations in the *GNPTAB*, *GNPTG*, *NAGPA* and *AP4E1* genes.^2^^,^^3^ This set of genes is known to be involved in transporting lysosomal enzymes from the endoplasmic reticulum to lysosomes.^4^ Homozygous loss-of-function mutations in *GNPTAB* and *GNPTG* were previously known to cause the lysosomal storage diseases, Mucolipidosis Types II and III,^4^^,^^5^ while homozygous loss-of-function mutations in *AP4E1* have been associated with spastic paraplegia and cerebral palsy.^6^^,^^7^ Patients with these mutations are characterized clinically by abnormal physical and cognitive development, including absent or delayed speech.^1^

Rare genetic variations in the four genes associated with stuttering (*GNPTAB, GNPTG, NAGPA* and *AP4E1*) were found in ∼20% of the unrelated cases of persistent stuttering whereas their incidences in the general population is <1%.^3^^,^^8^ Most of the variants identified in people who stutter are heterozygous missense mutations and not associated with any physical or cognitive abnormalities present in lysosomal storage diseases.^8^ On the other hand, accumulating neuroimaging evidence has shown that stuttering is associated with structural and functional anomalies in the brain regions involved in interhemispheric connections, language processing and speech–motor control.^9–18^ However, the connection between genetics and brain anomalies in people who stutter has not been established because genetic backgrounds of the participants in the previous neuroimaging studies were unknown.

In this case–control study, we used MRI to detect subtle neuroanatomical anomalies in a group of family members who all stutter due to the same genetic cause. They are all mutation carriers of the same two heterozygous mutations in *AP4E1* gene (c.1549G>A and c.2401G>A) which, along with other rare mutations in this gene, have been shown to be associated with persistent stuttering in our previous genetic study.^3^ The control group was seven age-matched, unrelated normally fluent Cameroonians who do not have any of the *AP4E1* variants or a history of stuttering. Two MRI techniques were used: (i) high-resolution T_1_-weighted images for the measurement of grey matter volume (GMV) in cortical and subcortical areas and (ii) Diffusion tensor imaging (DTI) for the estimation of the fractional anisotropy (FA), which reflects microstructural coherence in white matter. Based on microarray gene expression data obtained from the Allen Institute for Brain Sciences (AIBS), we expected that the most prominent differences would be found in the thalamus and the corpus callosum where the expression of *AP4E1* is the highest.^19^^,^^20^

To further demonstrate that the differences are associated with the mutations instead of effects specific to the Cameroonian family, we quantified the spatial relationship between the pattern of GMV anomalies and *AP4E1* expression levels across supratentorial brain regions. The gene expression data from the AIBS have been used as a proxy of genetic effects on different parts of the brain, revealing gene–brain relationships in several previous studies.^17^^,^^18^^,^^21–24^ For example, Grothe et al.^23^ demonstrated that amyloid deposition in patients with Alzheimer’s disease is correlated with the expression of genes coding for the amyloid precursor protein. If mutated *AP4E1* contributes to the neuroanatomical anomalies in the mutation carrier group, we would expect that the effects would be proportional to the levels of *AP4E1* expression, leading to a spatial association between the pattern of GMV differences and *AP4E1* expression in the brain.^17^ Additionally, this relationship should be weaker in other gene associated with stuttering (*GNPTAB*, *GNPTB* and *NAGPA*).

## Methods

### Standard protocol approvals, registrations and patient consents

All participants were enrolled with written informed consent under National Institutes of Health (NIH) protocol 97-DC-0057 (ClinicalTrials.gov Identifier: NCT00001604) approved by the NIH Central Nervous System Institutional Review Board. Seven *AP4E1* mutation carriers (five males and two females; mean age: 29.7 years; standard deviation: 7.7) with persistent neurodevelopmental stuttering from a large Cameroonian family were recruited.^3^ All of them are heterozygous for the same *AP4E1* mutation haplotype (c.1549G>A and c.2401G>A). Stuttering was diagnosed by speech pathologists using the Stuttering Severity Instrument Third Edition.^25^ All the carriers displayed at least 4% dysfluency rate. Seven unrelated male non-carriers with no history of stuttering served as controls (mean age: 33.1 years; standard deviation: 10.1 years). They were recruited in the greater District of Columbia area in the USA and had immigrated to the USA from Cameroon within 6 months prior to their MRI measurements. Dideoxy sequencing was performed on *AP4E1, GNPTAB*, *GNPTG* and *NAGPA* to ensure that none of them carry a mutation in the known genes associated with stuttering. For both *AP4E1* carriers who stutter and controls, their clinical history and physical examinations were conducted at the NIH Clinical Center, Bethesda, Maryland. Apart from persistent stuttering in the mutation carrier group, physical examinations were normal for all participants.

### Research procedures

MRI images were acquired on a Siemens 3 T MAGNETOM Skyra scanner with a 16-channel head coil at the NIH Clinical Center. Whole-brain T_1_-weighted images were collected using magnetization-prepared rapid gradient-echo sequence with the following parameters: Time of Echo (TE) = 1.76 ms, Time of Repetition (TR) = 5.1 ms, Flip Angle = 15°, Resolution = 0.98 × 0.98 × 1.0 mm. Seventy whole-brain diffusion-weighted images with *b* values of 300 or 1100 s/mm^2^ and 10 non-diffusion weighted volumes (*b*0) were acquired in two runs using the following parameters: 80 axial slices, TR = 11.9 s, TE = 91 ms, Flip Angle = 90°, GRAPPA acceleration factor = 2, Resolution = 2 mm isotropic.

Voxel-based morphometry (VBM) analysis was performed using the CAT12 toolbox (http://www.neuro.uni-jena.de/cat/) and DARTEL normalization algorithm to obtain voxel-wise GMV.^26^^,^^27^ Modulated GMV images were resampled to 1.5 mm isotropic voxels and spatially smoothed using a Gaussian kernel with a full-width half maximum (FWHM) of 6 mm. Voxels with mean grey matter probability less than 0.5 were excluded from further analysis. Group-level analysis of GMV was conducted using the General Linear Model (GLM) framework implemented by SPM12 (https://www.fil.ion.ucl.ac.uk/spm/software/spm12/). Age, sex and intracranial volume were included in the GLM as nuisance variables. Since there were only two female participants in the mutation carrier group, including sex variable is important for capturing sex-specific effects. The initial voxel-wise thresholds were set at *P* < 0.005, and family-wise error (FWE) was corrected at the cluster level using Gaussian random field theory, corresponding to FWE-corrected *P* < 0.05.

Diffusion images were preprocessed using MRtrix *dwibiascorrect* script and FSL *eddy* commands.^28^ MRtrix was used to estimate diffusion tensors and derive FA maps for each subject. The preprocessed FA maps were analysed using two complementary methods. First, we used FSL’s tract-based spatial statistics (TBSS) algorithm to project individual FA values greater than 0.25 to a pseudo-anatomical white matter skeleton.^29^ FSL’s nonparametric permutation tool *randomise* was used to determine significant group differences.^30^ Age and sex were included in the model as nuisance variables. Statistic threshold was set at FWE-corrected *P* < 0.05. In addition to tract-based analysis, we used a voxel-based analysis of FA to show the spatial extent of the group differences. Individual FA images were normalized, resampled to 2 × 2 × 2 mm resolution and spatially smoothed using a Gaussian kernel with a FWHM of 6 mm. Voxels with mean FA less than 0.25 were excluded from group analysis. SPM’s GLM with sex and age as nuisance variables was used for group analysis. Statistical significance threshold was set at FWE-corrected *P* < 0.05. We focussed on FA because most previous DTI studies on stuttering reported group differences in this measure.^31^ To complement the results of FA, we examined other DTI measures, including axial diffusivity (AD), radial diffusivity (RD) and mean diffusivity (MD).

### Association between *AP4E1* expression and GMV differences

Gene expression levels in different brain regions were obtained using the method previously described.^17^ Briefly, normalized microarray-based gene expression data from six adult donors (five males, one female; age: 24–57 years; see http://www.brain-map.org/ for details) were obtained from the AIBS.^19^^,^^20^ This data set contains expression of all protein coding genes in approximately 3700 samples from different brain regions. The anatomical locations of the samples were converted to the Montreal Neurological Institute (MNI) space by applying the transformation field obtained from DARTEL normalization of donor’s T_1_-weighted images using CAT12 toolbox. The expression of each gene from different probes was first averaged. The medians of all samples in each of the left cortical and subcortical regions defined by a standard atlas (AAL Atlas) were used to represent the overall expression level in the region.^32^ Regions in the right hemisphere were excluded because the majority of the samples were collected in the donors’ left hemispheres. The globus palladus was also excluded because it was consistently classified as white matter in all of our subjects. Similar to our previous study, regional GMV differences were obtained by averaging the magnitude of voxel-wise between-group *t*-statistics within each left supratentorial region defined by AAL Atlas.^17^ The association between the spatial patterns of *AP4E1* expression and GMV differences across regions was evaluated using Spearman’s correlation.^17^ In addition, this correlation analysis was conducted on the other three genes associated with stuttering (*GNPTAB*, *GNPTG* and *NAGPA*) to show that the spatial relationship was specific to *AP4E1*.

### Data availability statement

The datasets generated and/or analysed during the current study are available from the corresponding author upon request.

## Results

**Figure 1:**
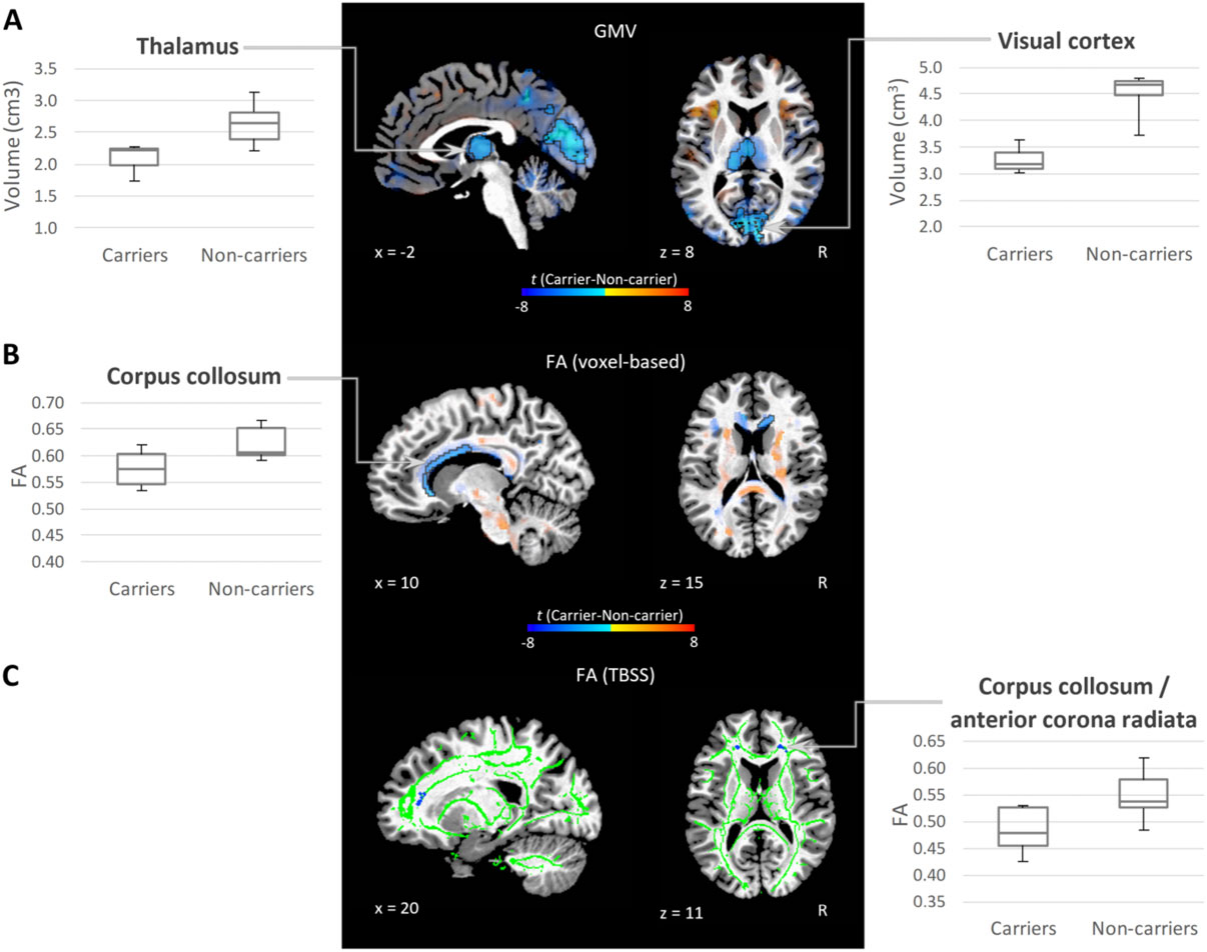
**Grey and white matter regions showed significant differences between carriers and non-carriers of *AP4E1* mutations**. (**A**) *T* statistics of the between-group differences in GMV and (**B**) FA are overlaid on a single subject template. Orange indicates that the neuroimaging measures are larger in *AP4E1* carriers than controls at uncorrected *P* < 0.05, while blue indicates the opposite. Areas that exhibited a significant group difference at corrected *P* < 0.05 are outlined by black lines. The *P*-values of the significant clusters are listed in Tables 1 and 2. Both voxel-wise group level analyses of GMV and FA were conducted using the GLM with group and sex as factors and age as a covariate. The analysis of GMV also included intracranial volume as a covariate. The box plot in each panel shows the median, minimum, maximum, first and third quartiles of the neuroimaging measure in a cluster exhibiting a significant difference between *AP4E1* carriers and controls. (**C**) Results of TBSS analysis of FA. Individual skeletonized FA was analysed using the same GLM of the voxel-wise FA analysis. Significant FA reductions in the *AP4E1* carriers relative to controls are indicated in blue and overlaid on a white matter skeleton (green) and a single subject template. No significant difference was found in the opposite direction. The significant clusters on the skeleton were dilated one voxel to increase their visibility.

**Figure 2:**
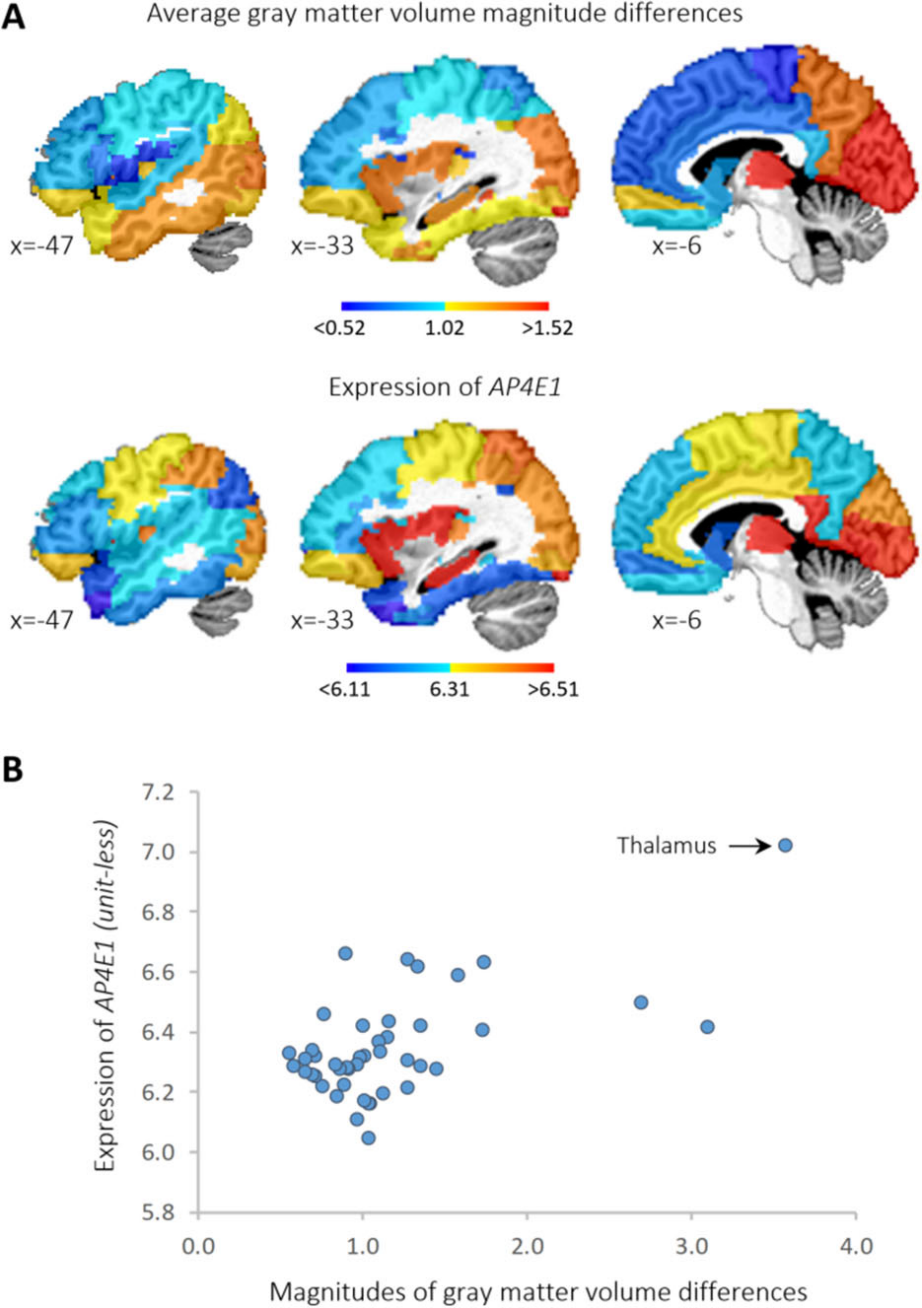
**Relationship between *AP4E1* expression and between-group GMV differences**. (**A**) Magnitudes of GMV differences and *AP4E1* expression in the left supratentorial regions defined by a standard atlas (AAL). (**B**) A scatter plot of regional *AP4E1* expression and between-group GMV differences. Each dot represents a supratentorial region defined by a standard atlas (AAL) in the left hemisphere.

We observed significant differences between *AP4E1* mutation carriers who stutter and non-carrier, normally fluent controls in both GMV (Fig. 1A) and white matter diffusivity measures (Fig. 1B and C). The anatomical locations of these differences are listed in Tables 1 and 2. Compared to non-carrier controls, *AP4E1* mutation carriers with persistent stuttering exhibited smaller GMV in the thalamus, the posterior cingulate gyrus and the calcarine gyrus (Fig. 1A). Furthermore, *AP4E1* mutation carriers showed decreased FA in the corpus callosum in both tract-based (TBSS) and voxel-based analyses (Fig. 1B and C). Additionally, *AP4E1* mutation carriers showed increased AD, RD and MD in the corpus collosum (Tables 1 and 2 and Supplementary Fig. 1). As expected, the regions that exhibited between-group differences in GMV and FA were the highly expressed locations of *AP4E1* in the brain (Supplementary Table 1). In particular, the thalamus and the corpus collosum were the regions showing the highest expression of *AP4E1* among the supratentorial regions defined by AAL atlas. Furthermore, the magnitude of between-group GMV differences and *AP4E1* expression were moderately correlated (*r_s_* = 0.38, *P* = 0.011) across supratentorial regions. This relationship is illustrated in Fig. 2. Since both *AP4E1* expression and GMV difference were markedly larger in the thalamus than the other regions, we repeated the same analysis without the thalamus to ensure that the correlation was not solely driven by the thalamus. In this follow-up analysis, the spatial association was still significant (*r_s_* = 0.34, *P* = 0.028), indicating that this relationship was not solely driven by the thalamus. Moreover, the spatial correlations between the patterns of GMV differences and expression patterns of the other three genes associated with stuttering were not significant (*GNPTAB*: *r_s_* = −0.15, *P* = 0.32, *GNPTG*: *r_s_* = 0.21, *P* = 0.16, *NAGPA*: *r_s_* = 0.14, *P* = 0.38), and are illustrated in Supplementary Fig. 2.

**Table 1:** The locations and spatial extent of the significant differences between *AP4E1* carriers and controls in the VBM and the voxel-based DTI analyses

Region/MRI measure/contrast	Hemisphere	Peak *x*, *y*, *z*	Size (cm^3^)	Corrected *P*
Grey matter volume—Carriers < Non-carriers				
Thalamus	L/R	−10, −30, 14	58.7	0.001
Visual cortex and posterior cingulate cortex	L/R	−2, −68, 14	147.7	<0.001
FA—Carriers < Non-carriers				
Genu and midbody of the corpus callosum	L/R	10, 24, −6	20.3	0.002
Axial diffusivity—Carriers > Non-carriers				
Genu, midbody and splenium of the corpus callosum	L/R	4, 4, 26	43.2	<0.001
Radial diffusivity—Carriers > Non-carriers				
Genu and midbody of the corpus callosum	L/R	2, 2, 24	77.1	<0.001
Mean diffusivity—Carriers > Non-carriers				
Genu and midbody of the corpus callosum	L/R	2, −6, 26	84.6	<0.001
Splenium of the corpus callosum	L	−24, −52, 10	20.6	0.023

**Table 2.** The locations and spatial extent of the significant differences between *AP4E1* carriers and controls in the tract-based analyses of DTI measures (TBSS)

Region/MRI measure/contrast	Hemisphere	Peak *x*, *y*, *z*	# voxel (1 mm^3^)	Corrected *P*
FA—Carriers < Non-carriers				
Genu of the corpus callosum/anterior corona radiata	R	21, 35, 12	40	0.050
Genu of the corpus callosum/anterior corona radiate	L	−20, 36, 12	13	0.050
Axial diffusivity—Carriers > Non-carriers				
Genu and midbody of the corpus callosum	L/R	13, 33, 8	4041	0.002
Splenium of the corpus callosum	L/R	−1, −38, 9	64	0.044
Radial diffusivity—Carriers > Non-carriers				
Genu of the corpus callosum/anterior corona radiata	L	−20, 35, 11	1392	0.032
Genu of the corpus callosum/anterior corona radiata	R	22, 33, 15	341	0.040
Mean diffusivity—Carriers > Non-carriers				
Genu and midbody of the corpus callosum	L/R	−4, 24, 14	7650	0.024
Splenium of the corpus callosum	R	19, −36, 31	322	0.042

## Discussion

The objective of this study was to examine the neuroanatomical effects of specific heterogeneous *AP4E1* mutations that were previously associated with persistent stuttering.^3^ We compared *AP4E1* mutation carriers who stutter from a Cameroonian family with a group of unrelated, age- and ethnicity-matched, normally fluent, non-carriers who had no mutations in their *AP4E1* gene. The ideal control group to compare with the carriers of *AP4E1* mutations would be normally fluent members of the same family who do not carry the mutations. However, these individuals were not available for our study. While we are unable to rule out family-specific effects that are unrelated to the mutations by using unrelated Cameroonians as controls, we believe that the observed neuroanatomical anomalies are likely to be associated with the *AP4E1* mutation because the patterns of between-group structural differences appeared to be congruent with the expression of *AP4E1* obtained from an independent sample. Specifically, not only were the largest between-group differences observed in brain regions where *AP4E1* are highly expressed, i.e. the thalamus and the corpus collosum, but also the pattern of GMV differences across supratentorial regions was associated with the expression levels of *AP4E1* in the brain. This congruency suggests that the structural differences between carriers and controls are potentially associated with these mutations in *AP4E1* that have previously been associated with stuttering.

It is important to note that gene expression data used in the correlation analysis were not obtained from our participants because it is not feasible to take their brain tissue samples. Our observed spatial correlation between GMV differences and expression of *AP4E1* does not imply a direct causal relationship between *AP4E1* mutations and the *AP4E1* expression levels in the carriers. Moreover, the gene expression data from AIBS are based only on six donors, and variability between donors may affect the representativeness of the data. However, up to now, the expression data from the AIBS represent the only comprehensive survey of gene expression in the human brain, and previous studies suggest that the patterns of gene expression in adult donors appear to exhibit a high degree of similarity.^19^^,^^20^^,^^33^

Are effects of *AP4E1* mutations different from or the same as other causes of stuttering? Previous neuroimaging studies on people who stutter may give us some insight on this question. These previous studies did not genotype their participants, and thus their mutation status was unknown. However, the incidence of *AP4E1* mutations in unrelated people who stutter is only 3.6%^1^ and we can assume that their contribution to the results of the previous neuroimaging studies is minimal. Thus, the previous results mostly reflect brain anomalies associated with mutations in genes other than *AP4E1*, environmental effects or other factors. Several small studies have investigated GMV in adults who stutter with unknown mutations status.^34–38^ These studies reported that GMV differences between adults who stutter and controls were located in the frontotemporal areas or the caudate nuclei. In contrast, in our study, GMV differences were observed most prominently in the thalamus and the calcarine gyrus. This discrepancy indicates that effects of *AP4E1* mutations on grey matter may be different from stuttering due to other causes and that different neural subtypes may exist in stuttering. This notion is further supported by a recent study in which the pattern of GMV differences between children who stutter (who were also not genotyped) and controls was shown to be significantly correlated with the expression of *GNPTG* and *NAGPA*, but not in *AP4E1*.^17^ However, the results of the current study should be interpreted cautiously because of its small sample size. Moreover, there were only two female participants in the mutation carrier group, which limits our ability to examine potential interactions between sex and the effects of *AP4E1* mutations. Larger imaging genetic studies are needed to confirm the unique and common effects of *AP4E1* and their interactions with other factors.

Although GMV differences in the thalamus were not indicated in the previous studies, abnormalities in the thalamus as a part of the basal ganglia thalamocortical (BGTC) network have long been suggested to be involved with stuttering.^39–41^ According to a biologically plausible neurocomputational model of speech production (GODIVA), the BGTC network is involved in an internalized timing mechanism that supports the initiation and sequencing of speech articulatory gestures.^42^^,^^43^ Anatomically, this process could be achieved through projections from the premotor areas to the basal ganglia, which further project to the supplementary motor area (SMA) and pre-SMA via the thalamus.^44^ In support of this, direct electrical stimulation during awake brain surgery has demonstrated that transient stuttering-like dysfluencies can be elicited by stimulating a single region in the components of BGTC network, including the thalamus.^45–49^ These previous studies indicate that deficits in the thalamus may potentially contribute to the symptoms of stuttering.

Interestingly, compared to non-carrier controls, *AP4E1* mutation carriers with persistent stuttering also exhibited smaller GMV in the calcarine gyrus and the posterior cingulate cortex. According to data from the AIBS, the expression of *AP4E1* in these two regions is relatively high (see Supplementary Table 1), indicating that the GMV decreases are likely to be associated with the mutations. However, they are not typically associated with speech production and their involvement in stuttering is unclear.

In addition to GMV differences, reduced FA in the corpus callosum was observed in the carriers of *AP4E1* mutations. It is consistent with a number of previous DTI studies of adults and children who stutter due to unknown causes.^9^^,^^14^^,^^15^^,^^50–52^ Moreover, a recent study showed that knock-in mice carrying *Gnptab* mutations, homologous to previously identified human stuttering mutations and functionally related to *AP4E1*, exhibited reduced astrocyte density and volume in the corpus callosum together with vocalization deficits similar to those in human stuttering.^53^ This animal study and our current study both indicate that structural abnormalities in the corpus callosum can be driven by specific genetic factors. However, the roles of the corpus collosum in speech production and stuttering are not fully understood. Perhaps, structural abnormalities in the corpus collosum could adversely affect hemispheric specialization of language,^54^ which have been hypothesized as a contributing factor in persistent stuttering.^11^

In conclusion, this study provides preliminary evidence on the neuroanatomical effects associated with the *AP4E1* mutations in people who stutter. Specifically, we showed that *AP4E1* mutations were associated with anomalies in the brain structures previously linked to persistent stuttering, including the thalamus and the corpus callosum.

## Supplementary material

Supplementary material is available at *Brain Communications* online.

## Supplementary Material

fcab266_Supplementary_Data

## Abbreviations

AIBS =: Allen Institute for Brain Sciences
DTI =: Diffusion tensor imaging
FA =: fractional anisotropy
GLM =: General Linear Model
GMV =: grey matter volume
VBM =: Voxel-based morphometry
