# Supplementary material for: Neuroanatomical anomalies associated with rare AP4E1 mutations in people who stutter
Source: Brain Commun. 2021 Nov 13;3(4):fcab266. doi: 10.1093/braincomms/fcab266 (PMC8633735; doi:10.1093/braincomms/fcab266)
Supplement: fcab266_Supplementary_Data [file fcab266_supplementary_data.pdf]

Supplementary Materials

Table S1. *AP4E1* expression levels obtained from Allen Institute for Brain Science in the left supratentorial brain regions defined by AAL brain atlas and the corpus callosum.

Region labels in AAL brain atlas	***AP4E1 Expression***
Thalamus_L	7.02
Corpus Callosum*	6.99
Pallidum_L	6.87
Cingulum_Post_L	6.66
Hippocampus_L	6.64
Amygdala_L	6.63
Insula_L	6.62
Lingual_L	6.59
Calcarine_L	6.50
Parietal_Sup_L	6.46
Heschl_L	6.44
Parietal_Inf_L	6.42
Occipital_Mid_L	6.42
Cuneus_L	6.42
Occipital_Sup_L	6.41
Occipital_Inf_L	6.38
Frontal_Inf_Orb_L	6.37
Cingulum_Mid_L	6.34
Frontal_Mid_Orb_L	6.34
Paracentral_Lobule_L	6.33
Cingulum_Ant_L	6.32
Precentral_L	6.32
Postcentral_L	6.32
Supp_Motor_Area_L	6.31
Temporal_Mid_L	6.30
Frontal_Mid_L	6.29
Rectus_L	6.29
Precuneus_L	6.29
Rolandic_Oper_L	6.29
SupraMarginal_L	6.28
Temporal_Sup_L	6.28
Olfactory_L	6.28
ParaHippocampal_L	6.27
Frontal_Sup_Medial_L	6.27
Putamen_L	6.26
Frontal_Inf_Oper_L	6.25

Frontal_Inf_Tri_L	6.22
Frontal_Sup_L	6.22
Temporal_Inf_L	6.21
Frontal_Medial_Orb_L	6.19
Caudate_L	6.18
Fusiform_L	6.17
Angular_L	6.16
Temporal_Pole_Sup_L	6.16
Frontal_Sup_Orb_L	6.11
Temporal_Pole_Mid_L	6.05

*AAL atlas only defines gray matter areas. The expression of *AP4E1* in the corpus callosum was based on the annotation of the donors' samples provided by the Allen Human Brain Atlas. Histological staining of tissue sections was used to identify and annotate donors' neuroanatomic structures, including the corpus callosum. For details, please see https://help.brain-map.org/download/attachments/2818165/WholeBrainMicroarray_WhitePaper.pdf.

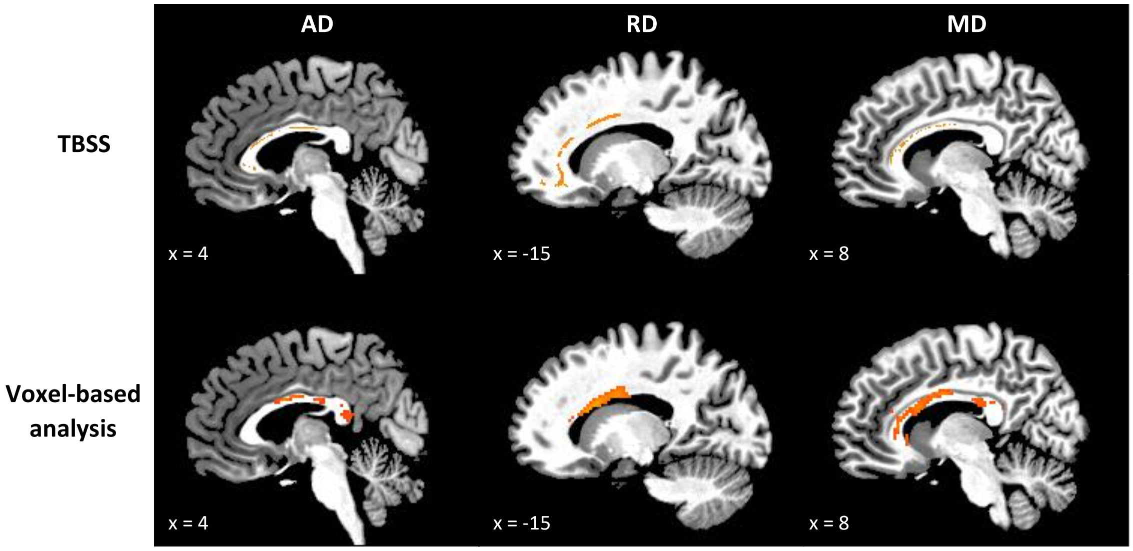

Figure S1. Results of tract-based (TBSS) and voxel-based analyses of axial diffusivity (AD), radial diffusivity (RD) and mean diffusivity (MD). Results are overlaid on a single subject template. Orange indicates that the measures were larger in *AP4E1* carriers who stutter than controls at corrected $p<0.05$. No significant difference was found in the opposite direction.

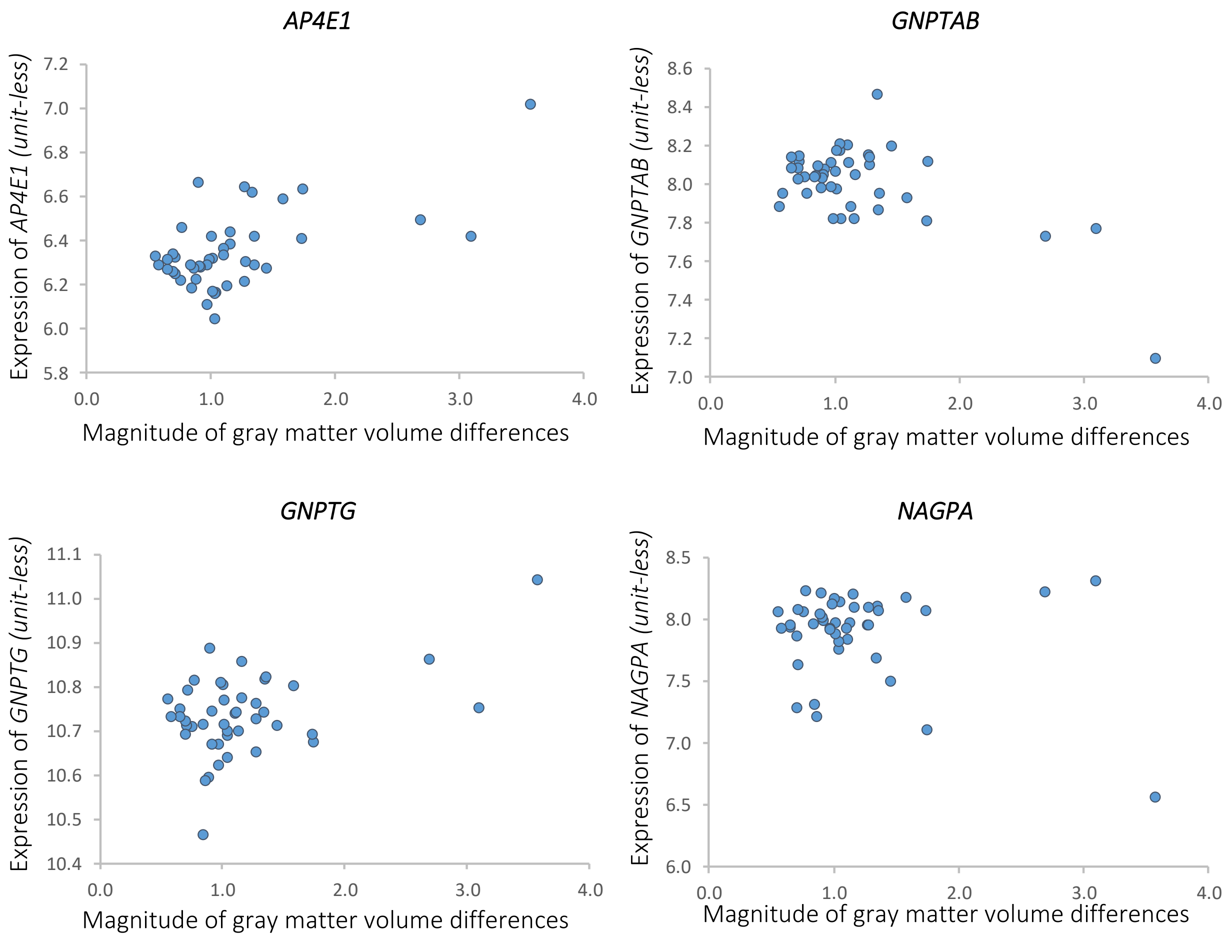

Figure S2. Relationship between expression of the four genes associated with stuttering and magnitudes of gray matter volume differences. Each dot represents a supratentorial region defined by a standard atlas (AAL) in the left hemisphere.
